# Adhesion-regulating molecule 1 (ADRM1) can be a potential biomarker and target for bladder cancer

**DOI:** 10.1038/s41598-023-41992-8

**Published:** 2023-09-08

**Authors:** Qing-xin Yu, Jiao-chen Wang, Jun-fei Liu, Lu-xia Ye, Yi-qing Guo, Hai-hong Zheng

**Affiliations:** 1 Department of Pathology, Taizhou Hospital, Wenzhou Medical University, Linhai, 317000 Zhejiang Province China; 2 Ningbo Clinical Pathology Diagnosis Center, Ningbo City, Zhejiang Province China

**Keywords:** Cancer, Oncology, Urology

## Abstract

Adhesion-regulating molecule 1 (ADRM1) has been implicated in tumor development, yet its specific role in bladder cancer (BC) remains undefined. This study aimed to elucidate the function of ADRM1 in BC through a combination of bioinformatics analysis and immunohistochemical analysis (IHC). Utilizing R version 3.6.3 and relevant packages, we analyzed online database data. Validation was conducted through IHC data, approved by the Institutional Ethics Committee (Approval No. K20220830). In both paired and unpaired comparisons, ADRM1 expression was significantly elevated in BC tissues compared to adjacent tissues, as evidenced by the results of TCGA dataset and IHC data. Patients with high ADRM1 expression had statistically worse overall survival than those with low ADRM1 expression in TCGA dataset, GSE32548 dataset, GSE32894 dataset, and IHC data. Functional analysis unveiled enrichment in immune-related pathways, and a robust positive correlation emerged between ADRM1 expression and pivotal immune checkpoints, including CD274, PDCD1, and PDCD1LG2. In tumor microenvironment, samples with the high ADRM1 expression contained statistical higher proportion of CD8 + T cells and Macrophage infiltration. Meanwhile, these high ADRM1-expressing samples displayed elevated tumor mutation burden scores and stemness indices, implying potential benefits from immunotherapy. Patients with low ADRM1 expression were sensitive to cisplatin, docetaxel, vinblastine, mitomycin C, and methotrexate. According to the findings from bioinformatics and IHC analyses, ADRM1 demonstrates prognostic significance for BC patients and holds predictive potential for both immunotherapy and chemotherapy responses. This underscores its role as a biomarker and therapeutic target in BC.

## Introduction

Bladder cancer (BC) stands as one of the most common malignancies affecting the urinary system^1,2^. In 2022 approximately 81,180 new cancer cases and 17,100 cancer deaths in the United States^2,3^. Of these, BC can clinically be divided into two subtypes: muscle-invasive bladder cancer (MIBC) and non-muscle-invasive bladder cancer (NMIBC), based on whether tumors invade the detrusor muscle^4,5^. Despite the continuous development of treatments, such as surgical interventions, Bacillus Calmette-Guerin (BCG), chemotherapy, and immunotherapy, the prognosis and treatment methods of BC are still far from satisfactory^6,7^. For instance, radical surgery (RC) is the standard treatment for MIBC. However, this therapy would bring heavy economic, physical, and mental burdens to patients^8,9^. Although experiencing a traumatic therapy, MIBC patients will recur rapidly following RC^9,10^. In addition to surgery, many patients are resistant to drugs (such as BCG, chemotherapy), which predict a poor prognosis of BC patients^11,12^. Meanwhile, some of the respondents to these treatments had to compelled to discontinue due to severe treatment-related adverse events^13^. Thus, enhancing the prognosis of BC patients has prompted researchers to explore new therapeutical targets and optimize the utilization of existing therapies through the identification of robust biomarkers^14^.

Adhesion-regulating molecule 1 (ADRM1), a ubiquitin receptor located on the 26S proteasome^15^, play a role in the cell adhesion, deubiquitination, and proteolysis^16^. Within the context of human cancers, deubiquitinating proteins are increasingly recognized as pivotal oncogenes or tumor suppressor genes across various malignancies^17^. Notably, recent studies have highlighted a correlation between elevated ADRM1 expression and unfavorable overall survival (OS) in distinct malignancies, such as hepatocellular carcinoma^18^, gastric cancer^19^, and breast cancer^20^. Specifically, inhibiting ADRM1 would suppress cancer cells growth in vitro^18,21^. Furthermore, ADRM1 also encodes RPN13, a critical ubiquitin receptor, and the application of inhibitors targeting the ADRM1/RPN13 interaction has been shown exert a substantial restraining effect on the amplification of ovarian cancer cell^22^. Remarkably, these inhibitors have displayed the capacity to induce a synergistic cytotoxic response in ovarian cancer cell in conjunction with cisplatin or doxorubicin^22^. In the tumor microenvironment of ovarian cancer, RPN13/ADRM1 inhibitors can reverse immunosuppression which effects attributed to myeloid-derived suppressor cells^23^. All these results underscore the potential of ADRM1 as a promising prognostic biomarker and a potential new therapeutic target. However, the role of ADRM1 in the context of BC remains relatively unexplored in the current literature.

Therefore, in this study, we aim to evaluate the correlation between ADRM1 and BC through a comprehensive approach involving bioinformatics analysis and immunohistochemical analysis (IHC). Specifically, our study pursues two primary objectives: firstly, to scrutinize the expression profile of ADRM1 in BC and its prognostic implications; secondly, to explore potential associations between ADRM1 expression, the immune microenvironment, and chemotherapy responsiveness. For validation the results of bioinformation analysis, we performed IHC using the BC samples in our institution, with ethical clearance from the appropriate committee.

## Materials and methods

### Online data acquisition

The Cancer Genome Atlas (www.gdc.cancer.gov, TCGA)^24^ repository provided both the expression and clinical data encompassing BC tissues and adjacent non-tumor tissues. Then, the ‘limma’ package was employed to compare ADRM1 expression between 19 adjacent non-tumor samples and 414 BC samples, as previous description^26^. At same time, an investigation into ADRM1 expression in pan-cancer was undertaken. As external validations, GSE32548 and GSE32894 datasets were extracted from the Gene Expression Omnibus (https://www.ncbi.nlm.nih.gov, GEO)^27^. To evaluated the prognostic value of ADRM1, Kaplan‒Meier curves was use to compare the survival outcome among the high and low ADRM1 expression groups within the TCGA, GSE32548, and GSE32894 datasets. Moreover, we extended this prognostic evaluation to subgroups within these datasets.

### Functional analysis

We conducted Gene Ontology (GO) enrichment analysis and the Kyoto Encyclopedia of Genes and Genomes (KEGG)^28–30^ enrichment analysis were performed to explore the potential function of ADRM1 in BC. The outcomes of these analyses, characterized by P values < 0.05 and Q values < 0.05, were presented through bubble plots. The ADRM1-related pathways were enriched using Gene Set Enrichment Analysis (GSEA) based on the REACTOME pathways. Pathways with P values < 0.05 and false discovery rate (FDR) < 25% were displayed. Meanwhile, GeneMANIA (www.genemania.org)^31^ was employed to screen the interacting proteins of ADRM1.

### Immune-related analysis and chemotherapy prediction

Considering the study report^23^ and functional results indicate that ADRM1 regulates immune environment, we explore the role of ADRM1 in immune-related analysis. Initially, we compared the expression of various immune checkpoints within high and low ADRM1 expression groups. We compared the expression profiles of immune cell marker genes, extracted from CellMarker 2.0^32^ and the study by Zhang et al.^33^, between the high and low ADRM1 expression groups. Then, a comparative analysis of the composition of infiltrated immune cells were compared between the low and high ADRM1 expression groups using the CIBERSORT algorithm. In order to corroborate the findings from CIBERSORT, the TIP (Tracking Tumor Immunophenotype) methodology was employed to evaluate the presence of infiltrating immune cells within each sample^34^. Furthermore, the tumor mutational burden (TMB) for each BC sample were calculated using the “maftools” package, based on data downloaded from TCGA database. The TMB scores of the low and high ADRM1 expression groups were subject to comparison. To gauge cellular stemness, an mRNA expression‐based stemness index (mRNAsi) was established through a one-class logistic regression machine learning algorithm^35^. Cellular stemness has been recognized to play a significant role in influencing the response to chemotherapy and immunotherapy^35,36^. Thus, we assessed the relationship between the mRNAsi score and ADRM1 expression^37,38^. A comparison of response rates between the high and low ADRM1 expression groups was conducted using data from the IMvigor210 trial (EGAD00001003977), encompassing patients who had all received anti-PD-L1 agent (atezolizumab). In chemotherapy prediction, the “pRRophetic” package was used to calculate the half-maximal inhibitory concentration (IC50), a key indicator of drug sensitivity. The IC50 scores were compared between the low and high ADRM1 expression groups.

### Patients and clinical specimens

Between 2017 and 2019, a total of 195 BC patients accepted surgery at our hospital were enrolled in this study. There were 195 BC samples and 34 adjacent non-tumor tissues collected from the enrolled patients. Inclusion criteria stipulated that patients were clinically diagnosed and with no other malignancy history, age less than 18 or postoperative survival period less than 30 days. Clinical features including age, sex, TNM stages and so on, as previous description^39^. Clinical stages were determined according to the criteria of the 8th edition of AJCC/UICC system (AJCC, American Joint Committee on Cancer /UICC, Union for International Cancer Control). Our study was approved by the Ethical Committee of our institution, and the ethics review(K20220830), and all participants provided written informed consent.

### Immunohistochemical analysis

IHC was performed on paraffin-embedded 3 μm sections. Following deparaffinization with xylene and rehydration with a gradient of alcohol, antigen retrieval was carried out through heating in citrate buffer. To quench endogenous peroxidase activity, 3% H_2_O_2_ was applied, followed by blocking with 5% BSA. Subsequently, the sections were incubated with a primary antibody against ADRM1 (diluted at 1:800, #17,054–1-AP, Proteintech, China) for 1.5 h at room temperature. After three washes, the sections were covered with HRP-conjugated secondary antibody at room temperature for 30 min. Thereafter, specimens were stained with 3′,3-diaminobenzidine tetrahydrochloride, counterstained with hematoxylin. After alcohol dehydration, xylene vitrification and neutral gum seal, sections were photographed using Leica 2500 microscope.

To semi-quantitatively represent the ADRM1 immunostaining results, evaluable sections were classified into four IHC scores according to the percentage of staining positive cells within the total cancerous cells: IHC score 0, 0% positive; IHC score 1, 1–10% positive; IHC score 2, 11–50% positive; IHC score 3, > 50% positive.

### Statistical analysis

According to the normality and quality of variances of the data, one-way ANOVA or the Mann‒Whitney U test was used to perform statistical analysis of three or more continuous variables. Quantitative data in two groups were compared using Student’s t test. All analyzed data are displayed as the standard deviation (SD). A *p* < 0.05 was considered significant for all analyses, which were performed using R version 3.6.3 and relative packages. ns, *p* ≥ 0.05; *, *p* < 0.05; **, *p* < 0.01; ***, *p* < 0.001.

### Ethical considerations

The authors are accountable for all aspects of the work in ensuring that questions related to the accuracy or integrity of any part of the work are appropriately investigated and resolved. The study was conducted in accordance with the Declaration of Helsinki (as revised in 2013). The study was approved by the ethics board of Taizhou Hospital, Wenzhou Medical University (K20220830), and informed consent was obtained from all patients.

## Results

### The basic data of ADRM1 in BC

**Figure 1:**
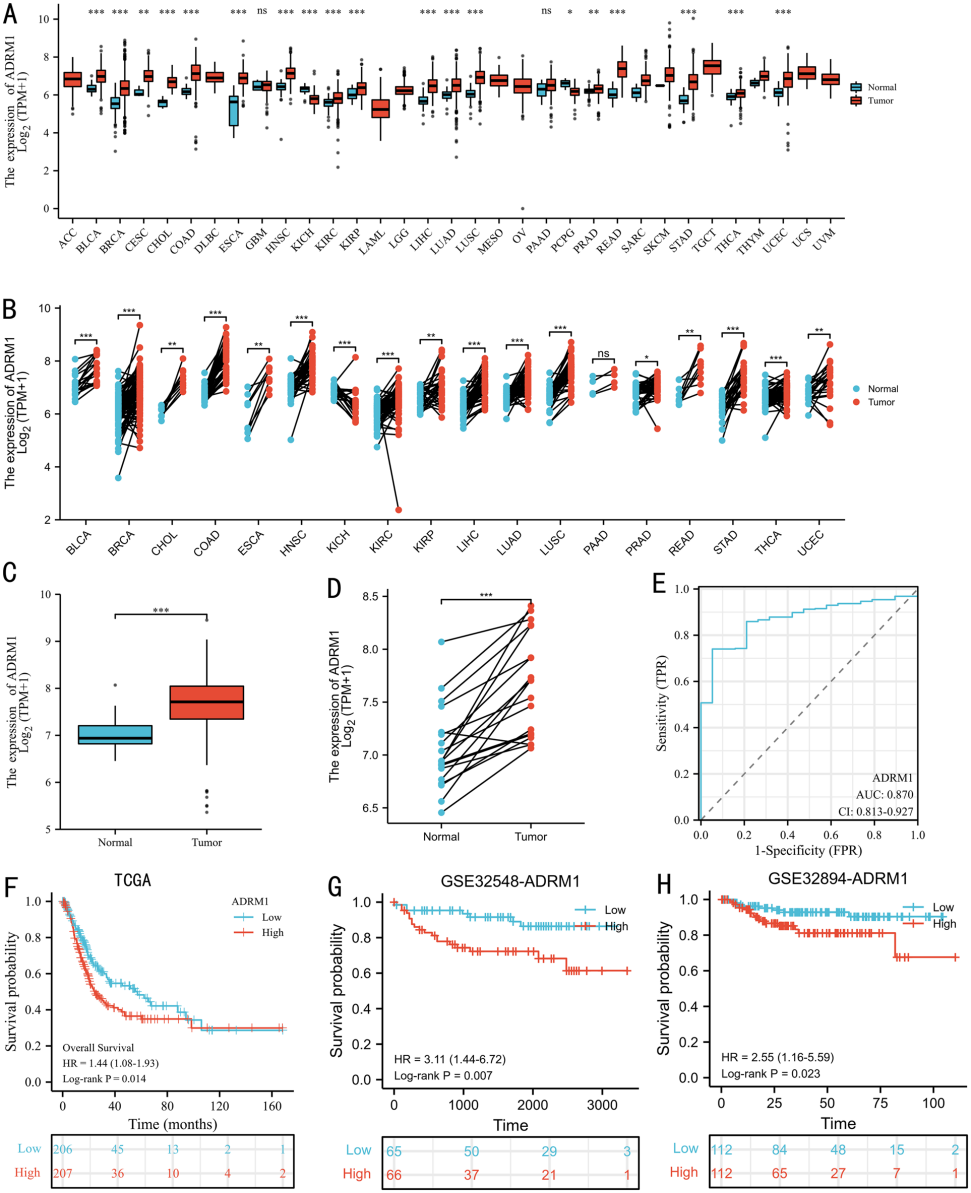
The expression of ADRM1 in unpaired (**A**) and paired (**B**) pan-cancer tissues. The expression of ADRM1 in unpaired (**C**) and paired (**D**) BC tissues. The diagnostic value of ADRM1 (**E**). The prognostic value of ADRM1 in TCGA (**F**), GSE32548 (**G**), and GSE32894 (**H**) datasets. ns, *p* ≥ 0.05; *, *p* < 0.05; **, *p* < 0.01; ***, *p* < 0.001.

As shown in Fig. 1A, B, ADRM1 was highly expressed in many cancers than counterpart normal tissues in both unpaired and paired samples, except kidney chromophobe, pheochromocytoma, and paraganglioma. Notably, the analysis revealed a statistically significant upregulation of ADRM1 expression in BC tissues compared to normal tissues, as depicted in both unpaired and paired comparisons (Fig. 1C, D).

Utilizing the ROC curve analysis, ADRM1 exhibited a moderately robust area under the curve (AUC) value of 0.87 (Fig. 1E). According to the median ADRM1 expression values extracted from the TCGA, GSE32548, and GSE32894 datasets, BC patients were divided into a high ADRM1 expression group (above the median) and a low ADRM1 expression group (below the median), respectively. As shown in Table 1, high ADRM1 expression was significantly associated with worse OS (*p* = 0.029) than low ADRM1 expression in the TCGA dataset. The detail information of GSE32548 and GSE32894 datasets were displayed in Supplementary Tables 1 and 2, respectively.

**Table 1:** The clinicopathological characteristics of the TCGA included bladder cancer patients.

Characteristic	ADRM1 mRNA expression		*p*
	Low, n (%)	High, n (%)	
n	207	207	
Age, mean ± SD	67.95 ± 10.97	68.13 ± 10.17	0.86
Sex, n (%)			1
Female	54 (13%)	55 (13.3%)	
Male	153 (37%)	152 (36.7%)	
T stage, n (%)			0.965
T1	3 (0.8%)	2 (0.5%)	
T2	59 (15.5%)	60 (15.8%)	
T3	94 (24.7%)	102 (26.8%)	
T4	29 (7.6%)	31 (8.2%)	
Distant metastasis, n (%)			0.86
M0	107 (50.2%)	95 (44.6%)	
M1	5 (2.3%)	6 (2.8%)	
AJCC stage, n (%)			0.428
Stage I	3 (0.7%)	1 (0.2%)	
Stage II	70 (17%)	60 (14.6%)	
Stage III	71 (17.2%)	71 (17.2%)	
Stage IV	62 (15%)	74 (18%)	
WHO grade, n (%)			0.646
High Grade	197 (47.9%)	193 (47%)	
Low Grade	9 (2.2%)	12 (2.9%)	
Overall survival, n (%)			0.029
Alive	127 (30.7%)	104 (25.1%)	
Dead	80 (19.3%)	103 (24.9%)	

*AJCC* American Joint Committee on cancer, *SD* Standard deviation, *WHO* World Health Organization, *n* Number.

### ADRM1predictes the prognosis of BC patients

**Figure 2:**
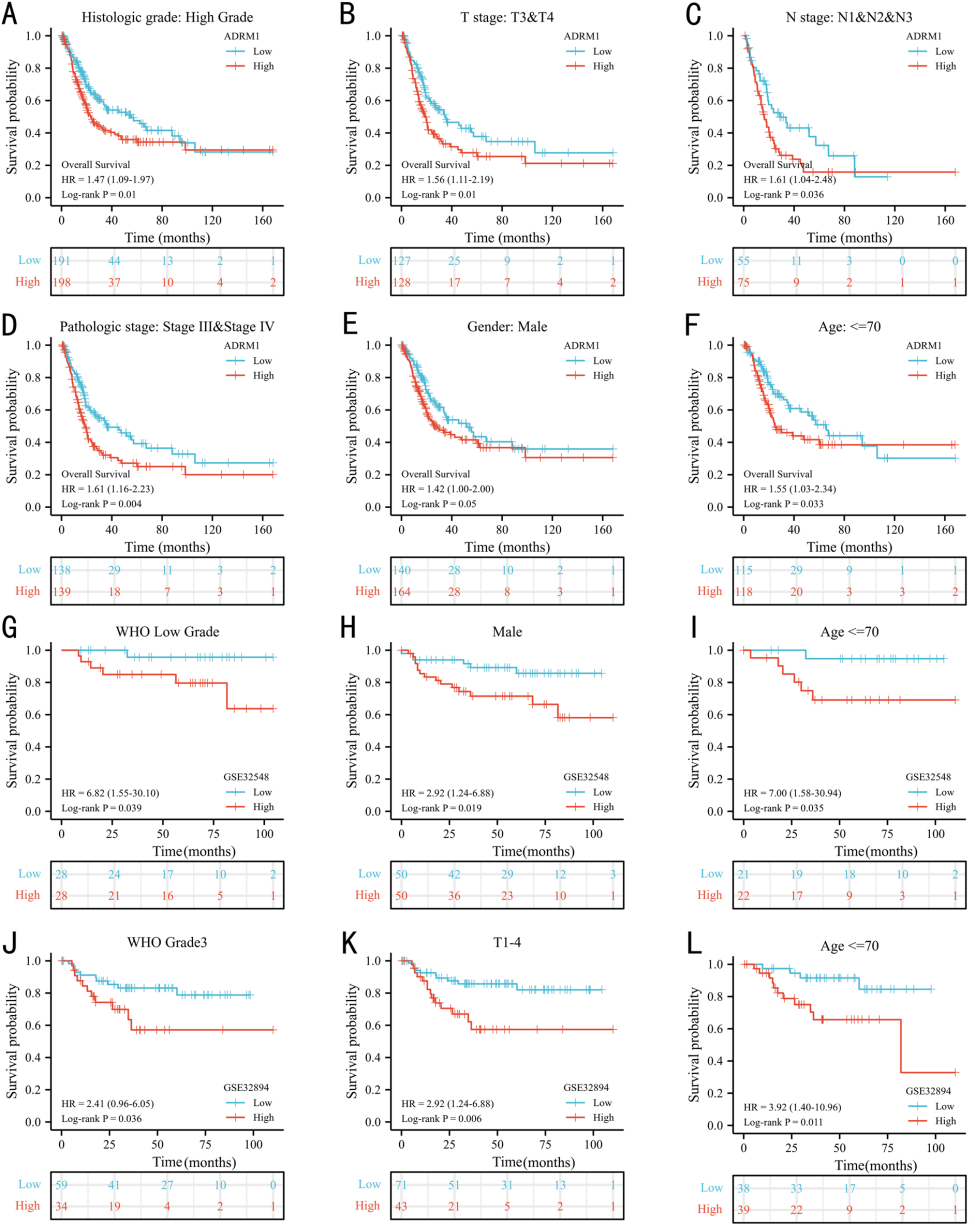
The prognostic value of ADRM1: TCGA subgroups [WHO high grade (**A**), T3_4 stage (**B**), lymph node metastasis (**C**), pathological stage (**D**), Male (**E**), and age <  = 70 years (**F**)]; GSE32584 subgroups [WHO low grade (**G**), male (**H**), and age <  = 70 years (**I**)]. GSE32894 subgroups [WHO G3 (**J**), T1_4 (**K**), and age <  = 70 years (**L**)].

The Kaplan–Meier curves clearly indicate a significant association between high ADRM1 expression and poor OS in BC patients (Fig. 1F, *p*  = 0.014) in the TCGA dataset. In external validations, patients with high ADRM1 expression also had shorter survival time than those with low ADRM1 expression in the GSE32548 (Fig. 1G, *p* = 0.007) and GSE32894 (Fig. 1H, *p* = 0.023) cohorts. Subgroup analyses also underscore this correlation, revealing high ADRM1 expression as significantly linked to adverse OS outcomes in several subgroups: World Health Organization (WHO) high grade (Fig. 2A, *p* = 0.01), T3-4 stage (Fig. 2B, *p* = 0.01), N1-3 stage (Fig. 2C, *p* = 0.036), pathological stage III-IV (Fig. 2D, *p* = 0.004), male patients (Fig. 2E, *p* = 0.05), and patients below 70 years old (Fig. 2F, *p* = 0.033).

In the external GSE32548 dataset, compared with the low ADRM1 expression group, the high ADRM1 expression group was significantly correlated with shorter OS in subgroups: WHO low grade (Fig. 2G, *p* = 0.039), male patients (Fig. 2H, *p* = 0.019) and patients below 70 years old (Fig. 2I, *p* = 0.035). In external GSE32984 dataset, ADRM1 also presented significant prognostic value in subgroups, such as WHO G3 (Fig. 2J, *p* = 0.036), T1_4 (Fig. 2K, *p* = 0.006) and patients below 70 years old (Fig. 2L, *p* = 0.011). These results revealed that ADRM1 expression might be a prognostic factor for BC patients.

### ADRM1 involves in immune-related pathways

**Figure 3:**
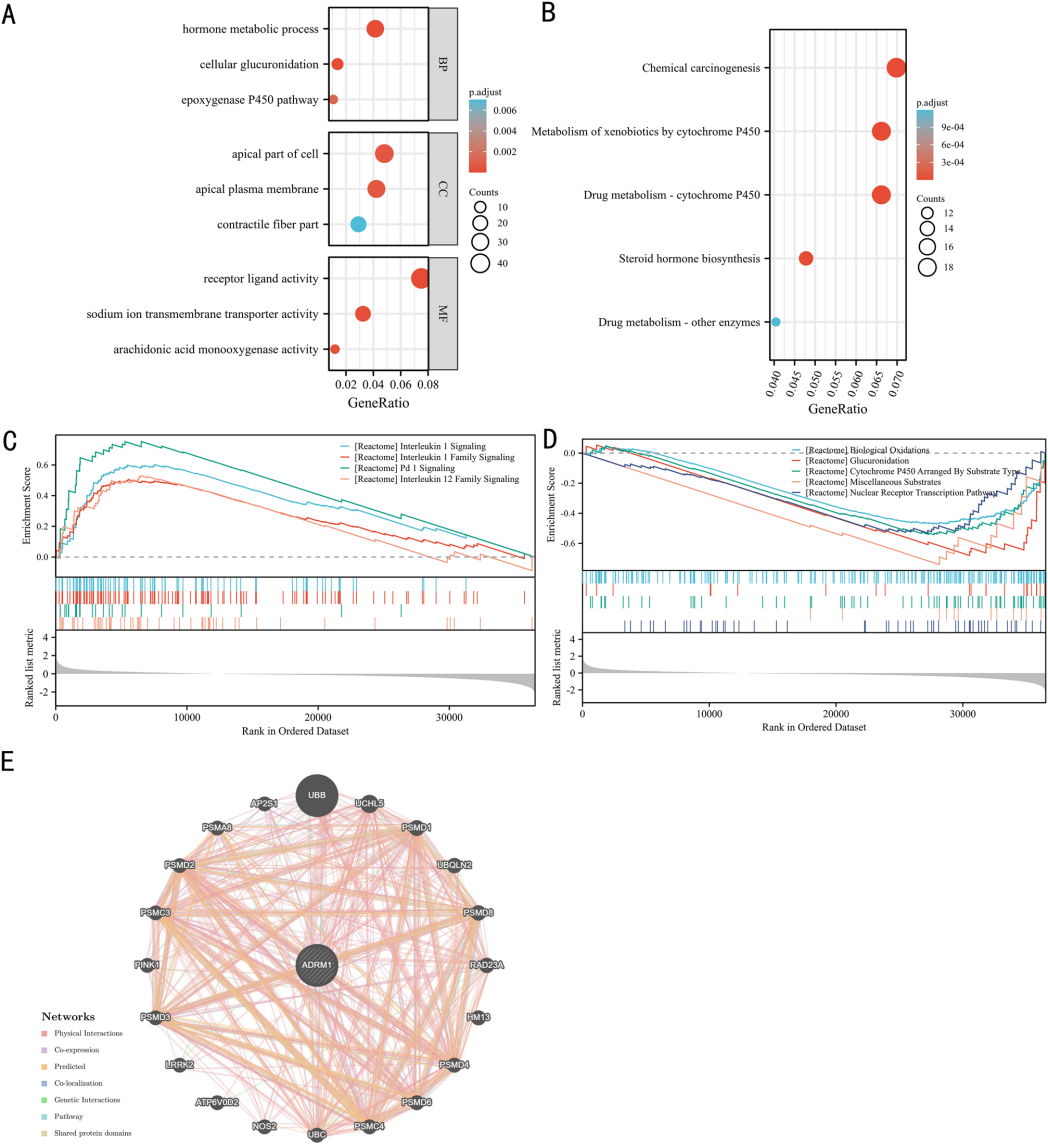
The results of functional analysis: the Gene Ontology results (**A**), Kyoto Encyclopedia of Genes and Genomes results (**B**), Gene Set Enrichment Analysis results (**C**, **D**), the protein–protein interaction network (**E**).

To further explore the potential function of ADRM1 in BC, a series of functional analyses performed utilizing ADRM1 differential genes based on the TCGA dataset. The GO enrichment analysis highlighted ADRM1's significant involvement in hormone metabolic process, apical part of cell, apical plasma membrane, receptor ligand activity and others (Fig. 3A). The KEGG pathway analysis underscored ADRM1's enrichment in pathways such as chemical carcinogenesis, metabolism of xenobiotics by cytochrome P450, and so on (Fig. 3B).

Subsequently, a GSEA analysis was conducted to discern the REACTOME pathways that exhibited differential regulation based on ADRM1 differentially expressed genes within the high and low ADRM1 expression groups. Figure 3C showed that ADRM1 differentially expressed genes were found to enriched immune-related categories, such as interleukin 1 signaling, interleukin 1 family signaling, PD1 signaling, and interleukin 12 family signaling pathways. Meanwhile, the GSEA revealed enrichments in processes such as biological oxidations, glucuronidation, miscellaneous substrates and nuclear receptor transcription pathways (Fig. 3D). According to the GeneMANIA results, we found that ADRM1 protein was associated with specific proteins such as UBB, UBC, NOS2, and PSMA8 (Fig. 3E).

### The immunological role of ADRM1 and ADRM1 predicts the response of chemotherapeutic drugs

**Figure 4:**
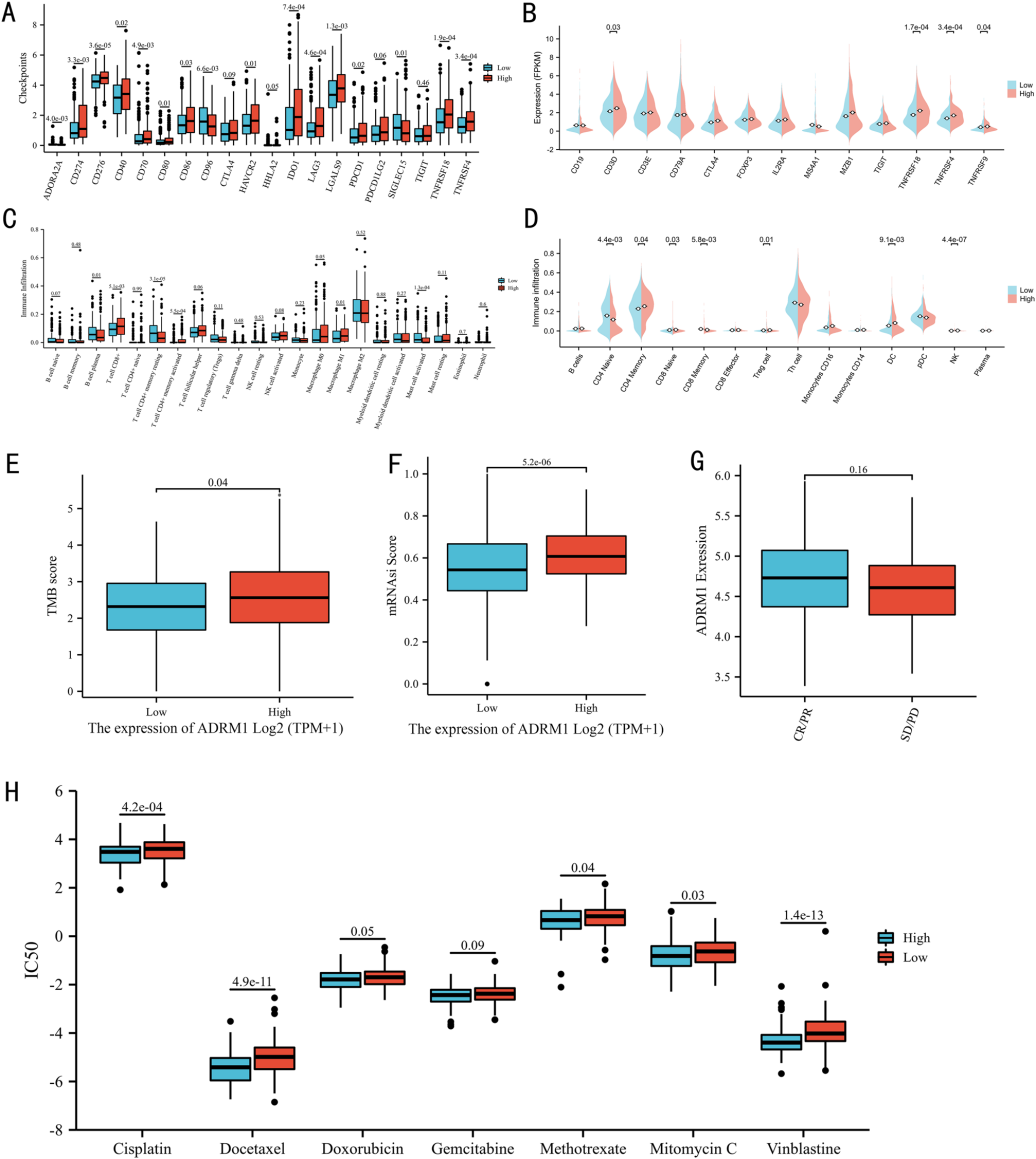
Immune-related analysis based on TCGA dataset: immune checkpoints (**A**), cell marker (**B**), immune infiltration on CIBERSORT (**C**) and TIP (**D**), the tumor mutation burden between high and low ADRM1 expression groups (**E**), the stemness index between high and low ADRM1 expression groups (**F**). The immunotherapy response in IMvigor210 (**G**). The IC50 of chemotherapy between the low and high ADRM1 groups (**H**). IC50: half-maximal inhibitory concentration.

The GSEA analysis prominently highlighted numerous gene sets enriched immune-related pathways, prompting a deeper investigation into the relationship between ADRM1 and immunological processes within the context of BC, utilizing data from the TCGA database. In high ADRM1 expression group, the expression of some important immune checkpoints increased, such as CD274 (PD-L1), CTLA4, PDCD1 (PD-1) and CDCD1LG2 (PD-L2) (Fig. 4A). The expression of cell markers notably demonstrated a substantial association between heightened ADRM1 expression and increased T cell infiltration within the high ADRM1 expression group (Fig. 4B). As the results unveiled a markedly elevated proportion of CD8 + T cells and M1 Macrophage in the high ADRM1 expression group, whereas a comparatively higher abundance of B plasma cell, CD4 + T memory resting cells and activated Mast cells was presented in the low ADRM1 expression group (Fig. 4C). In a parallel vein, the TIP analysis corroborated these findings, highlighting a significantly augmented proportion of CD8 + T naïve cells and CD4 + memory cells within the high ADRM1 expression group (Fig. 4D).

According to the low and high ADRM1 expression groups, we compared TMB score of BC. It's probably worth noting that patients in the high ADRM1 expression group had higher TMB score than those in the low ADRM1 expression group (Fig. 4E, p = 0.039). To explore mRNAsi in BC, we compared the expression of mRNAsi between the low and high ADRM1 expression groups. As shown, a significant difference in stemness indices within the low and high ADRM1 expression group, which suggested that the high ADRM1 expression group exhibited higher stemness than the low ADRM1 expression group (Fig. 4F, p < 0.001).

Patients with response had higher mean ADRM1 expression, whereas it did not reach a significant result (Fig. 4G). As the results illustrated that the IC50 values of cisplatin, docetaxel, vinblastine, mitomycin C and methotrexate in the high ADRM1 expression group were lower than that in the low ADRM1 expression group (Fig. 4H). These suggested that high expression of ADRM1 might poorly respond to chemotherapy.

### ADRM1 is a biomarker in real-world data

**Figure 5:**
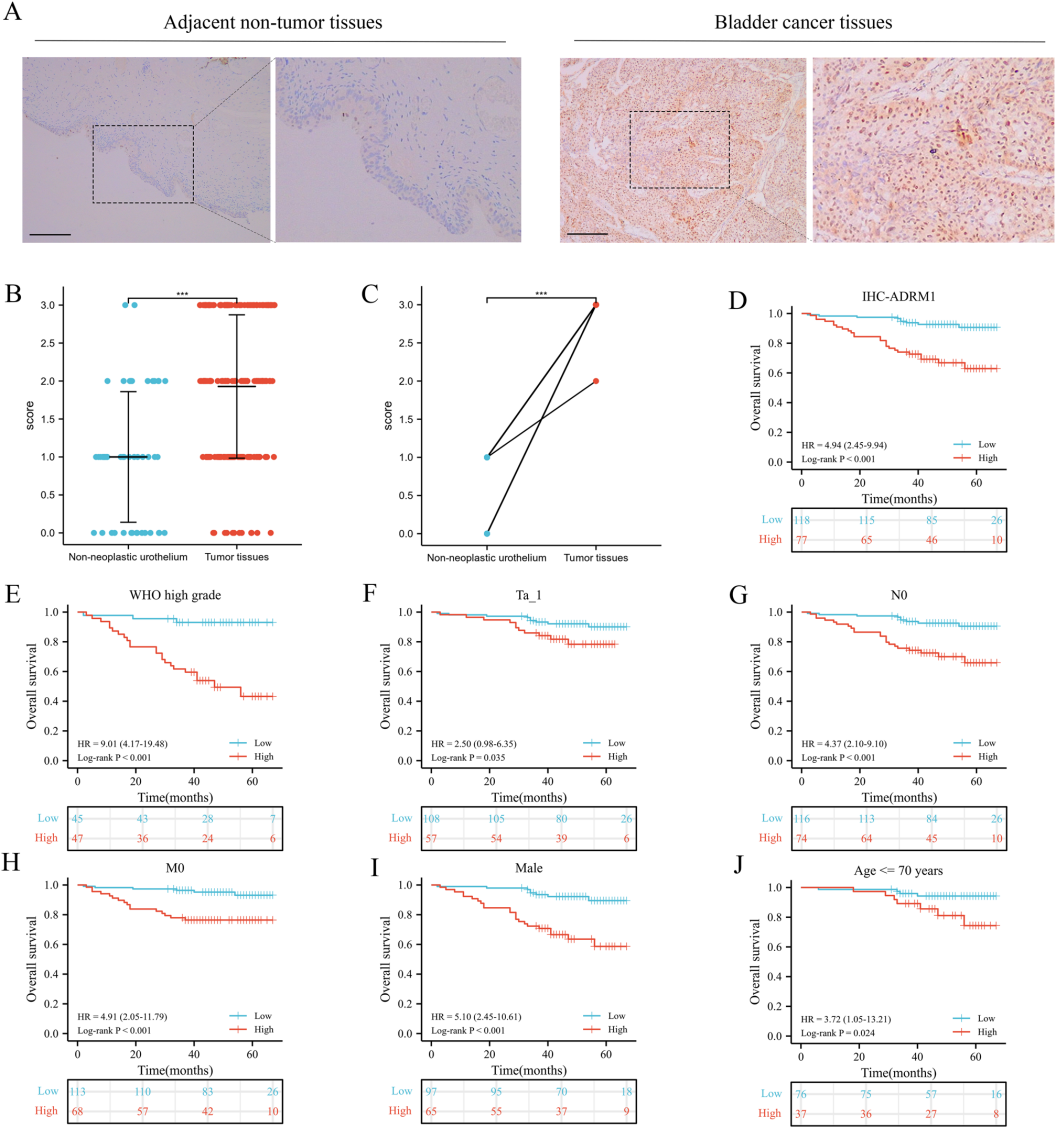
Validation the bioinformatics results of ADRM1: representative IHC staining for ADRM1 in BC and adjacent non-tumor tissues (**A**); the expression of ADRM1 in unpaired (**B**) and paired (**C**) tissues; the prognostic value of ADRM1 in IHC data: overall survival (**D**), subgroups [WHO high grade (**E**), Ta_1 stage (**F**), no lymph node metastasis (**G**), no distant metastasis (**H**), male (**I**), and age <  = 70 years (**J**)]; WHO: World Health Organization, IHC: Immunohistochemical analysis. ns, *p* ≥ 0.05; *, *p* < 0.05; **, *p* < 0.01; ***, *p* < 0.001.

Considering the previously observed elevation of ADRM1 mRNA in breast cancer (BC) patients, we extended our investigation to evaluate ADRM1 protein expression. Immunohistochemistry was conducted on tumor tissues from BC patients, and ADRM1 protein levels were assessed and scored in both tumor and adjacent non-tumor tissues. ADRM1 was predominantly localized within the nuclei of cancer cells. In the non-neoplastic surrounding urothelium, immunostaining for ADRM1 protein was mostly absent or exhibited weak intensity in most instances. (Fig. 5A). Immunostaining revealed higher protein expression for ADRM1 in most of the tumor tissues of BC patients in both unpaired (Fig. 5B) and paired (Fig. 5C) comparisons.

Based on the immunostaining intensity for ADRM1 in the tumor tissues, patients were categorized into low ADRM1 group (including patients scored by 0–2) and high ADRM1 group (including patients scored by 3). As shown in Table 2, the expression level of ADRM1 protein was observed to be associated with age, WHO grade, T stage and OS (Table 2).

**Table 2:** The clinicopathological characteristics of included bladder cancer patients from our institution.

Characteristic	ADRM1 IHC expression		*p*
	Low, n (%)	High, n (%)	
n	118	77	
Age, mean ± SD	66.33 ± 11.79	70.65 ± 10.23	0.009
Sex, n (%)			0.836
Female	21 (10.8%)	12 (6.2%)	
Male	97 (49.7%)	65 (33.3%)	
WHO grade, n (%)			0.003
Low grade	73 (37.4%)	30 (15.4%)	
High grade	45 (23.1%)	47 (24.1%)	
T stage, n (%)			0.013
Ta	82 (42.1%)	40 (20.5%)	
T1	26 (13.3%)	17 (8.7%)	
T2	5 (2.6%)	6 (3.1%)	
T3	2 (1%)	7 (3.6%)	
T4	3 (1.5%)	7 (3.6%)	
Lymph node metastasis, n (%)			0.385
N0	116 (59.5%)	74 (37.9%)	
N+	2 (1%)	3 (1.5%)	
Distant metastasis, n (%)			0.092
M0	113 (57.9%)	68 (34.9%)	
M1	5 (2.6%)	9 (4.6%)	
Overall survival, n (%)			< 0.001
Alive	109 (55.9%)	52 (26.7%)	
Dead	9 (4.6%)	25 (12.8%)	

*SD* Standard deviation, *WHO* World Health Organization, *n* Number.

As shown in Fig. 5D, Kaplan–Meier survival curves demonstrated that the high ADRM1 group had a shorter survival time than the low ADRM1 group (*p* < 0.001). To further examine the correlation between the expression of ADRM1 protein level and prognosis, Kaplan–Meier survival curves was employed to evaluated the prognostic value of ADRM1 in subgroups. In subgroups, ADRM1 also presented significant prognostic value in WHO high grade (Fig. 5E, p < 0.001), Ta_1 (Fig. 5F, p = 0.035), no lymph node metastasis (Fig. 5G, p < 0.001), no distant metastasis (Fig. 5H, p < 0.001), male (Fig. 5I, p < 0.001) and patients below 70 years old (Fig. 5J, p = 0.024). Collectively, the data from our institution corroborates the prognostic value of ADRM1 in predicting outcomes for BC patients.

## Discussion

ADRM1 is a ubiquitin receptor on the 26S proteasome, which is activated by the binding of ubiquitin and S1 subunit to the 19S complex^15^. This complex recruits the deubiquitinating enzyme UCH37 to the 26S proteasome^40^. In tumors, the 12 prognosis-related genes including ADRM1 might be promising therapeutic targets esophageal adenocarcinoma^41^. RA190, a specific inhibitor of ADRM1, induces apoptosis intrahepatic cholangiocarcinoma cells^42^. Moreover, ADRM1 interference combined with 5-fluorouracil treatment efficiently suppressed colorectal cancer cell growth in *vitro*^21^. These data support that ADRM1 may be essential for the maintenance of the malignant status of cells and thus affects the prognosis and treatment effect. Hence, we systematically investigated the role of ADRM1 in BC.

In this study, we investigated the relationship between ADRM1 expression and clinicopathological parameters and patient survival outcomes based on the TCGA database and two independent GEO datasets. The results revealed that ADRM1 mRNA significantly overexpressed in many tumors, including BC. Meanwhile, we adopted IHC to validate the results, which also identified that ADRM1 was highly expressed in BC tissues. Furthermore, high ADRM1 expression was associated with worse OS in BC patients. Subgroup analysis of both TCGA group and independent GEO datasets showed that a high ADRM1 expression was significantly correlated with a poor prognosis in BC in versus clinical subgroups. In our IHC data, ADRM1 predicted prognosis in whole cohort or subgroup of BC patients. Similarly, a study based on bioinformatics research also reported that a group of genes including ADRM1, PPARD, CST4, CSNK1E, PTPN14 and ETV6 could be a potential biomarker group for BC^43^. These results supported the idea that ADRM1 indeed be involved in the pathogenesis of BC, and it is more likely to be highly expressed of ADRM1 often predicts a poor prognosis.

We then applied function analysis, GO, KEGG, and GSEA, to explore the possible functions and mechanisms of action of ADRM1. Immune-related pathways were highly associated with the high ADRM1 expression group. According to the GeneMANIA results, we found that ADRM1 proteins were associated with proteins like UBB, UBC, NOS2, PSMA8 and PSMD2. UBB and UBC are two ubiquitin gene, which code for poly-Ub precursors. UBB or UBC protein was required for some cancer cells to keep tumor features, like myeloma cells^44^. NOS2 is an adaptive immune pathway gene, together with CD8A, CD68 and GZMB, show significant positive correlation with most of immune checkpoint coding genes in hepatocellular carcinoma^45^. PSMA8 is a member of PSMA family. The PSMA family genes were positively correlated with the cell cycle, ubiquinone metabolism, and immune response signaling^46^. PSMD2 expression was reported be correlated with immune cell infiltration in lung adenocarcinoma^46^. All these results shown that ADRM1 is highly likely to be an immune-related gene.

As the development of checkpoint inhibitor therapy, five immunotherapy agents targeting the primarily targeting programmed cell death-1 protein (PD-1) or its ligand (PD-L1) pathway have been approved by the US Food and Drug Administration (FDA) for BC patients, including atezolizumab, pembrolizumab, avelumab, nivolumab, and durvalumab. A study showed higher response rates with atezolizumab in patients with increased PD-L1 expression, compared to those with lower levels of PD-L1 expression^46^. In high ADRM1 expression group, the expression of PD-L1 and PD-1 increased, suggesting that patients with high ADRM1 expression were more likely to benefit from anti-PD-L1 therapy. The tumor microenvironment plays a vital role in immunotherapy^47,48^. In the aspect of infiltrating immune cells, the high ADRM1 group contained significantly higher proportion of CD8 + T cells and Macrophage (M1), and lower proportion of CD4 + T memory cells and Mast cells. There has reported that macrophage M1 was a predictor of immune-checkpoint blockades therapy for metastatic urothelial cancer patients^25,49^. Upregulated immune activation pathways observed in the high-M1 subset, which identified favorable response to immunotherapy^49^. Jia Lv et al. reported that BC patients with higher infiltration levels of CD8 + T cell and lower Mast cells are more likely to present with better immunotherapeutic effect and prognosis^50–52^. According to these data, we may infer that BC patients with high ADRM1 might has represent a better respond to immunotherapy than low ADRM1 patients. The high and low ADRM1 expression groups showed significant differences in TMB analysis. Higher ratio of relevant genomic alterations was found in the high ADRM1 expression group. And the expression of ADRM1 showed positive correlation with TMB score, which suggested that patients with different expression level of ADRM1 might suitable for different treatment options. Similarly, the high ADRM1 expression group was significantly correlated with higher stemness index than the low ADRM1 group. Patients with high stemness index were positively response to immunotherapy^35^. Thus, the results of immune-related analysis suggested that BC patients with high ADRM1 might has represent a better respond to immunotherapy than low ADRM1 patients.

Recently, a study reported that inhibition of ADRM1/RPN13 could has a synergistic cytotoxic response to ovarian cancer cell with cisplatin or doxorubicin^22^. Chen et al. also found that ADRM1 interference combined with 5-fluorouracil treatment efficiently suppressed colorectal cancer cell growth in vitro^21^. Hence, we evaluated the efficiency of chemotherapy in high and low ADRM1 expression groups. Consistent with the literatures, this research found that patients in the low ADRM1 expression group were more sensitive to multiple chemotherapy drugs. Given the results of immune-related analysis, patients could choose immunotherapy or chemotherapy as optimal treatment according to the level of ADRM1. Thus, we suggest that ADRM1 may be a useful biomarker for BC patients.

## Conclusion

By bioinformatics and IHC analyses, we identified that ADRM1 had prognostic value in BC patients and could predict the immunotherapy and chemotherapy responses, indicating that it is a biomarker and target of BC. Of course, many basic and animal experiments are required to further identified these results, which also is the next work of us.

### Supplementary Information

Supplementary Table S1.

Supplementary Table S2.

## Data Availability

Since human data are involved, you should contact the corresponding authors to obtain relevant information.
