# Supplementary material for: Adhesion-regulating molecule 1 (ADRM1) can be a potential biomarker and target for bladder cancer
Source: Sci Rep. 2023 Sep 8;13:14803. doi: 10.1038/s41598-023-41992-8 (PMC10491834; doi:10.1038/s41598-023-41992-8)
Supplement: Supplementary file 1 — Supplementary Table S1. [file 41598_2023_41992_MOESM1_ESM.docx]

Supplementary table 1. Correlation between ADRM1 expression and the clinicopathological features of bladder cancer patients in GSE32548 dataset.

| Characteristic | ADRM1 mRNA expression | | p |
| --- | --- | --- | --- |
|  | Low, n (%) | High, n (%) |  |
| n | 65 | 66 |  |
| Age, mean ± SD | 70.35 ± 11.24 | 69.23 ± 10.01 | 0.546 |
| Sex, n (%) |  |  | 0.961 |
| Female | 16 (12.2%) | 15 (11.5%) |  |
| Male | 49 (37.4%) | 51 (38.9%) |  |
| WHO grade, n (%) |  |  | 0.006 |
| Low grade | 36 (27.5%) | 20 (15.3%) |  |
| High grade | 29 (22.1%) | 46 (35.1%) |  |
| Overall survival, n (%) |  |  | 0.018 |
| Alive | 58 (44.3%) | 47 (35.9%) |  |
| Dead | 7 (5.3%) | 19 (14.5%) |  |

SD: Standard deviation; WHO: World Health Organization; n: Number.
