# Supplementary material for: Adhesion-regulating molecule 1 (ADRM1) can be a potential biomarker and target for bladder cancer
Source: Sci Rep. 2023 Sep 8;13:14803. doi: 10.1038/s41598-023-41992-8 (PMC10491834; doi:10.1038/s41598-023-41992-8)
Supplement: Supplementary file 2 — Supplementary Table S2. [file 41598_2023_41992_MOESM2_ESM.docx]

Supplementary table 2. Correlation between ADRM1 expression and the clinicopathological features of bladder cancer patients in GSE32894 dataset.

| Characteristic | ADRM1 mRNA expression | | p |
| --- | --- | --- | --- |
|  | Low, n (%) | High, n (%) |  |
| n | 112 | 112 |  |
| Age, mean ± SD | 68.29 ± 10.58 | 70.57 ± 11.88 | 0.131 |
| Sex, n (%) |  |  | 1 |
| Female | 31 (13.8%) | 30 (13.4%) |  |
| Male | 81 (36.2%) | 82 (36.6%) |  |
| WHO grade, n (%) |  |  | 0.096 |
| G1 | 17 (7.7%) | 28 (12.6%) |  |
| G2 | 40 (18%) | 44 (19.8%) |  |
| G3 | 53 (23.9%) | 40 (18%) |  |
| T stage, n (%) |  |  | 0.073 |
| T1 | 31 (13.8%) | 32 (14.3%) |  |
| T2 | 28 (12.5%) | 15 (6.7%) |  |
| T3 | 5 (2.2%) | 2 (0.9%) |  |
| T4 | 1 (0.4%) | 0 (0%) |  |
| Tx | 47 (21%) | 63 (28.1%) |  |
| Overall survival, n (%) |  |  | 0.09 |
| Alive | 95 (42.4%) | 104 (46.4%) |  |
| Dead | 17 (7.6%) | 8 (3.6%) |  |

SD: Standard deviation; WHO: World Health Organization; n: Number.
